# Tenofovir Modulates Semaphorin 4D Signaling and Regulates Bone Homeostasis, Which Can Be Counteracted by Dipyridamole and Adenosine A2A Receptor

**DOI:** 10.3390/ijms222111490

**Published:** 2021-10-25

**Authors:** Patricia Llamas-Granda, Laura Martin-Rodríguez, Raquel Largo, Gabriel Herrero-Beaumont, Aránzazu Mediero

**Affiliations:** Bone and Joint Research Unit, IIS-Fundación Jiménez Díaz UAM, 28040 Madrid, Spain; Patricia.llamas@icloud.com (P.L.-G.); laura.martinrodriguez@estudiante.uam.es (L.M.-R.); RLargo@fjd.es (R.L.); GHerrero@fjd.es (G.H.-B.)

**Keywords:** Sema4D, tenofovir, bone turnover, HIV, adenosine A2A receptor, osteoblast

## Abstract

Semaphorin 4D (Sema4D) is a neurotrophin that is secreted by osteoclasts and binds to its receptor PlexinB1 on osteoblasts to inhibit their differentiation and function. Adenosine A2A activation inhibits osteoclast Sema4D-mediated secretion, diminishes inflammatory osteolysis and prevents bone loss following tenofovir (one of the most used antivirals in HIV). Therefore, tenofovir might activate Sema4D signaling to alter bone turnover. Female C57Bl/6/A2AKO mice were ovariectomized and treated with saline (control), tenofovir 75 mg/Kg/day, dipyridamole 25 mg/Kg/day or a combination for 5 weeks and long bones were prepared for histology. Primary murine-induced osteoclast/osteoblast were challenged with tenofovir/dipyridamole 1 μM each, and the expression of Sema4D/PlexinB1, RhoA/ROCK/IGF1R was studied by RT-PCR, Western blot and immunostaining. In vivo tenofovir showed an increased expression of Sema4D when compared to control mice, and dipyridamole reverted the expression in an A2A-dependent manner. In vitro, tenofovir increases Sema4D expression and secretion in osteoclast precursors, and pre-treatment with dipyridamole reverted this effect. pRhoA and ROCK1 activation were increased and IRS1/IGF1R expression was diminished by tenofovir in the Vav3/ARHGAP18 mechanism in osteoblast precursors and reverted by dipyridamole in an A2A-dependent manner. This suggests that tenofovir increases bone loss by activation of Sema4D/PlexinB1 signaling, which inhibits osteoblast differentiation. Agents that increase local adenosine concentrations, such as dipyridamole, might prevent bone loss following the inhibition of this pathway.

## 1. Introduction

One of the main mechanisms for maintaining bone homeostasis is communication between osteoclasts and osteoblasts. Among the variety of molecules that mediate this communication, we can find Semaphorins, members of the family of axonal guidance proteins [1,2,3]. Semaphorin 4D (Sema4D)/CD100 is secreted by osteoclasts in the presence of RANKL and binds to its receptor PlexinB1 on osteoblasts to inhibit their differentiation and function by activating RhoA/ROCK, which inhibits insulin-like growth factor-1 (IGF-1) signaling [4,5,6].

Sema4D was first identified as a regulator of the immune response as it mediates cell-cell interactions during immune synapses [7]. Sema4D is constitutively expressed in CD4 and CD8 T cells [8], and has low expression in resting B cells and antigen-presenting cells, and it is up-regulated in these cells upon activation [9]. Sema4D and its receptor PlexinB1 inhibit the migration of monocytic and B-cell lineage cells [10]. Reciprocally, when Sema4D acts as a receptor, it regulates T cell activation [11]. Sema4D is also expressed, although to a much lesser extent, on macrophages, NK, dendritic cells, B cells and neutrophils [12]. A soluble form of Sema4D (sCD100) upregulates cytokines (IL-2, IL-6, TNFα among others) [13] and leads to the regulation of dendritic cells and B cells [14]. Recently, Sema4D is elevated in the sera of patients with several infectious and inflammatory disorders [8,15].

It has been recently found that loss of Sema4D expression plays an important role in dysfunctional immunity in HIV infection [16,17]. It has recently been observed that chronic HIV patients under successful highly active antiretroviral therapy (HAART) have low levels of sCD100, despite their CD4 and CD8 cell counts [18].

Tenofovir, an AMP analog and one of the most commonly used antivirals in HIV, increases bone catabolism markers and decreased bone mineral density (BMD) [19]. Recent data have demonstrated that tenofovir blocks ATP release by Pannexin-1 [20], and Pannexin-1 hemichannel and ATP signaling are essential for HIV entry and replication in CD4+ T lymphocytes [21]. We have recently demonstrated that tenofovir, via a decrease in extracellular ATP and adenosine levels, increases osteoclast differentiation and function both in vitro and in vivo, reduces bone production and bone volume, and inhibits bone formation in vivo [22]. In the same work, we observed that treatment with agents that increase local adenosine concentrations, such as dipyridamole, an agent that blocks the adenosine taken up by the cells, prevents bone loss following tenofovir treatment depending on intact adenosine A2A receptor signaling [23]. Previously, in the laboratory, we found that adenosine A2A receptor activation diminished the secretion of Sema4D by osteoclasts and diminished bone osteolysis [24].

As Sema4D is a key modulator of bone turnover and plays an important role in HIV immunity, and knowing that tenofovir diminished BMD in HIV patients, we, therefore, hypothesize that Sema4D signaling might be activated under tenofovir treatment, and increasing extracellular adenosine concentration with dipyridamole may counteract this effect by the activation of adenosine A2A receptor.

## 2. Results

### 2.1. Mice Treated with Tenofovir Showed an Increased Expression of Sema4D

**Figure 1:**
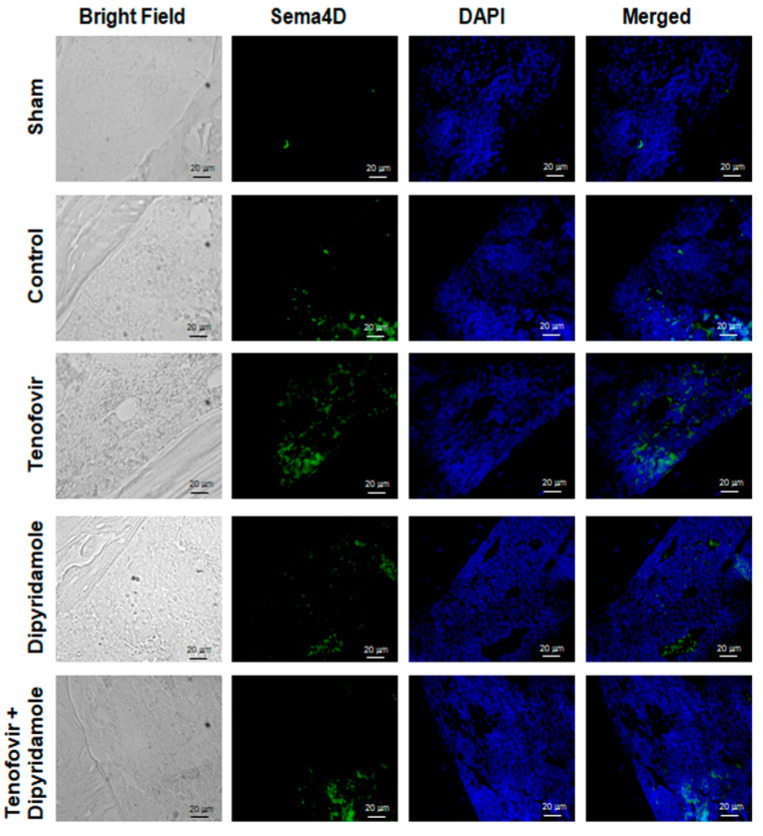
OVX mice treated with tenofovir showed an increased expression of Sema4D. Long bones were processed, and immunohistologic staining was carried out. Representative images are stained for Sema4D (green); Nuclei are shown in blue (DAPI). All images were taken at 40× magnification and scale bar indicates 20 µm segment.

**Figure 2:**
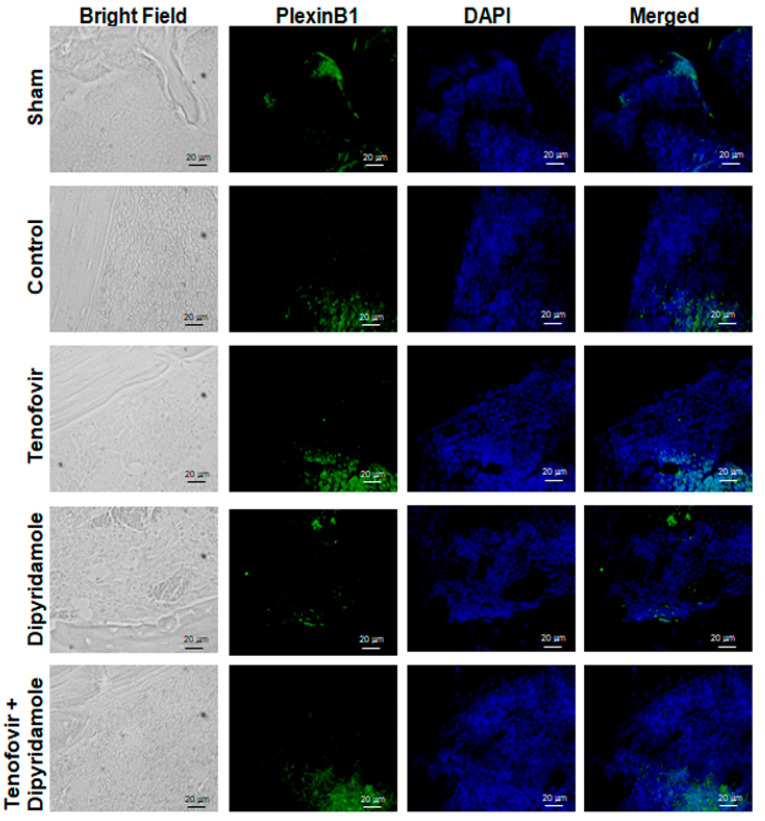
OVX mice treated with tenofovir showed no changes in PlexinB1. Long bones were processed and immunohistologic staining was carried out. Representative images stained for PlexinB1 (green); Nuclei are shown in blue (Dapi). All images were taken at 40× magnification and scale bar indicates 20 µm segment.

In order to explore the involvement of Sema4D signaling in Tenofovir bone effects, we performed inmunostaining analysis in femur in ovarectomize (OVX) wild-type mice. After treating WT OVX mice for 5 weeks with tenofovir, we observed an increasing number in Sema4D-positive cells, and these positive cells were reverted in the presence of dipyridamole (Figure 1). Nevertheless, we did not see any change in the number of PlexinB1-positive cells among the different treated mice (Figure 2).

### 2.2. Tenofovir Increased Sema4D Expression and Secretion In Vitro

**Figure 3:**
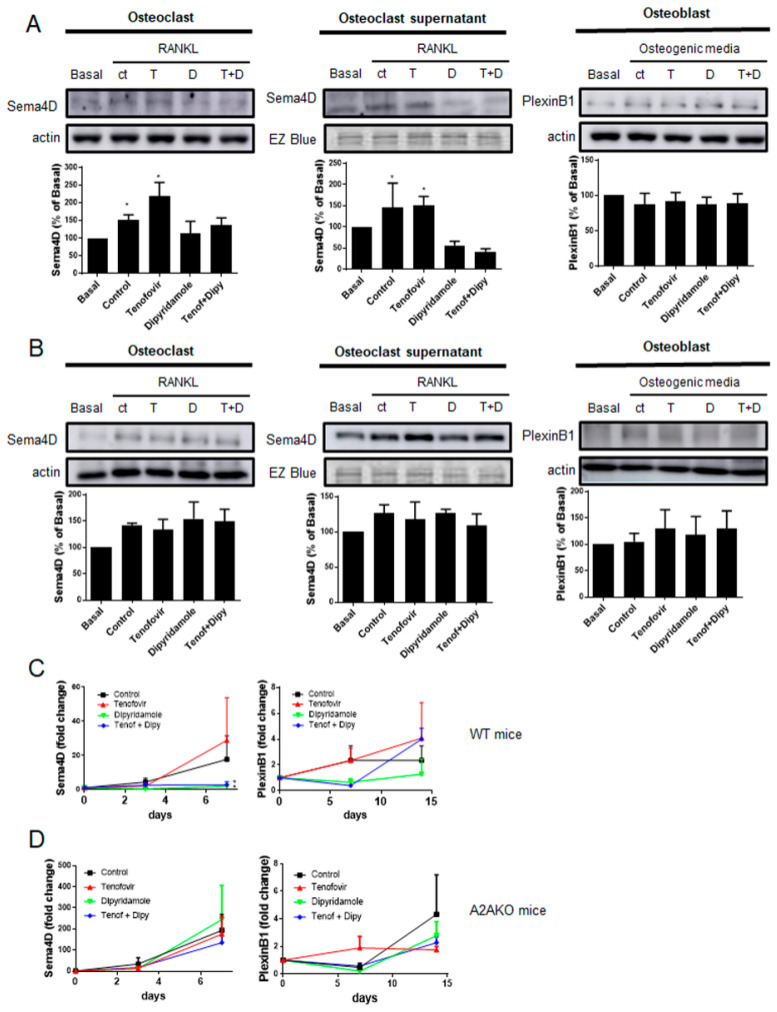
Sema4D expression and secretion are increased in the presence of tenofovir, and they are reverted with dipyridamole in an A2A-dependent manner. (**A**) Sema4D protein expression and secretion were studied in WT osteoclast precursors in the presence of tenofovir at 1 μM alone or with dipyridamole at 1 μM for 24 h. PlexinB1 protein expression was studied in WT osteoblast precursors in the presence of tenofovir at 1 μM alone or with dipyridamole at 1 μM for 24 h. To normalize for protein loading, the membranes were reprobed with actin or EZ Blue (supernatant). (**B**) Sema4D protein expression and secretion were studied in A2AKO osteoclast precursors in the presence of tenofovir at 1 μM alone or with dipyridamole at 1 μM for 24 h. PlexinB1 protein expression was studied in A2AKO osteoblast precursors in the presence of tenofovir at 1 μM alone or with dipyridamole at 1 μM for 24 h. To normalize for protein loading, the membranes were reprobed with actin or EZ Blue (supernatant). (**C**) Changes in Sema4D and PlexinB1 mRNA during osteoclast and osteoblast differentiation process respectively in WT mice in the presence of tenofovir 1 μM alone or with dipyridamole 1 μM. (**D**) Changes in Sema4D and PlexinB1 mRNA during osteoclast and osteoblast differentiation processes, respectively, in A2AKO mice in the presence of tenofovir 1 μM alone or with dipyridamole 1 μM. * *p* < 0.05 ANOVA.

The in vivo results show that tenofovir might be able to promote Sema4D expression from osteoclasts. To corroborate this fact, Sema4D expression and secretion were evaluated in vitro by Western blotting. As described, 24 h after RANKL stimulation, Sema4D expression and secretion were increased (51 ± 14% and 33 ± 26 increased vs. basal, *p* < 0.05, respectively) (Figure 3A). Tenofovir at 1 μM increased Sema4D expression (119 ± 32% increased vs. basal, *p* < 0.05) and secretion (51 ± 20% increased vs. basal, *p* < 0.05) 24 h after stimulation in osteoclast precursors, and pre-treatment with dipyridamole at 1 μM reverted this effect (Figure 3A). When Plexin B1 expression was analyzed, no changes were observed at the protein level 24 h after osteoblast precursors were challenged with any compound when compared to basal or control levels (Figure 3A). When we analyzed A2AKO osteoclast precursors, we observed an increased expression and secretion when compared to basal level in all treatment conditions (Figure 3B) and no changes in PlexinB1 receptor with any treatment (Figure 3B).

Similar results were observed at the mRNA level. During osteoclast differentiation, tenofovir at 1 μM increased Sema4D expression (up to 28-fold change) in a similar manner as to control with RANKL alone (up to 18-fold change) with no significant differences among them, and this was completely abrogated with dipyridamole at 1 μM (*p* < 0.05) (Figure 3C). PlexinB1 mRNA expression increased during osteoblast differentiation but there were no significant changes in the presence of any treatment (Figure 3C). Sema4D mRNA expression was increased in all the treatment conditions in A2AKO during osteoclast differentiation (Figure 3D), and no changes in Plexin B1 were observed (Figure 3D).

### 2.3. Phosphorylation of RhoA and ROCK1 Activation Were Increased by Tenofovir in Osteoblasts

**Figure 4:**
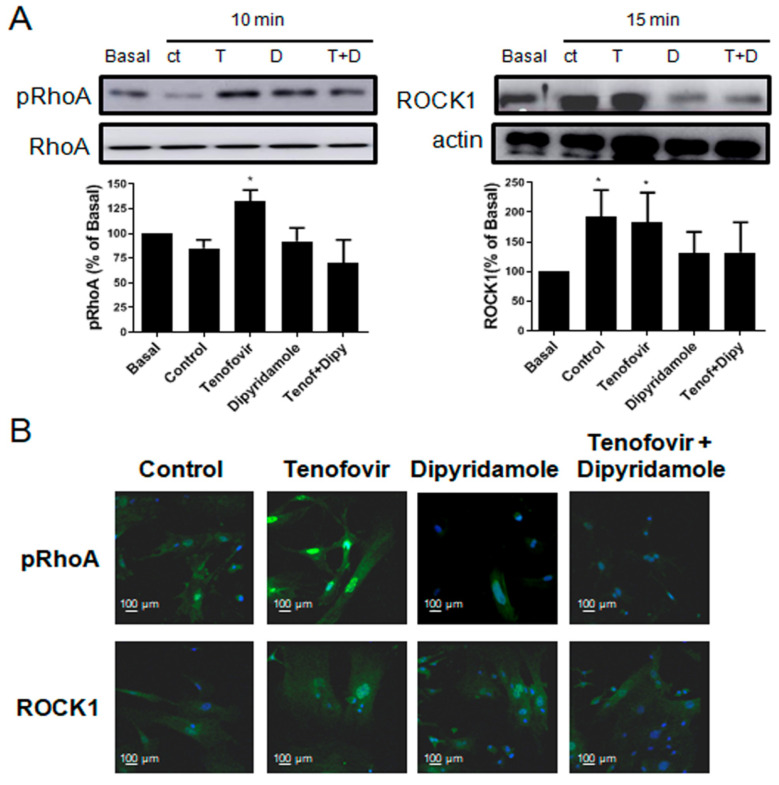
Phosphorylation of RhoA and ROCK1 activation were increased by tenofovir in osteoblast in WT **mice.** (**A**) pRhoA and ROCK1 expression were studied in osteoblast precursors in the presence of tenofovir at 1 μM alone or with dipyridamole at 1 μM. To normalize for protein loading, the membranes were reprobed with RhoA or actin. (**B**) pRhoA and ROCK1 (green) immunostaining in osteoblast precursors treated with tenofovir at 1 μM alone or with dipyridamole at 1 μM. Nuclei are shown in blue (Dapi). All images were taken at 40× magnification, and scale bar indicates 100 µm. * *p* < 0.05 ANOVA.

**Figure 5:**
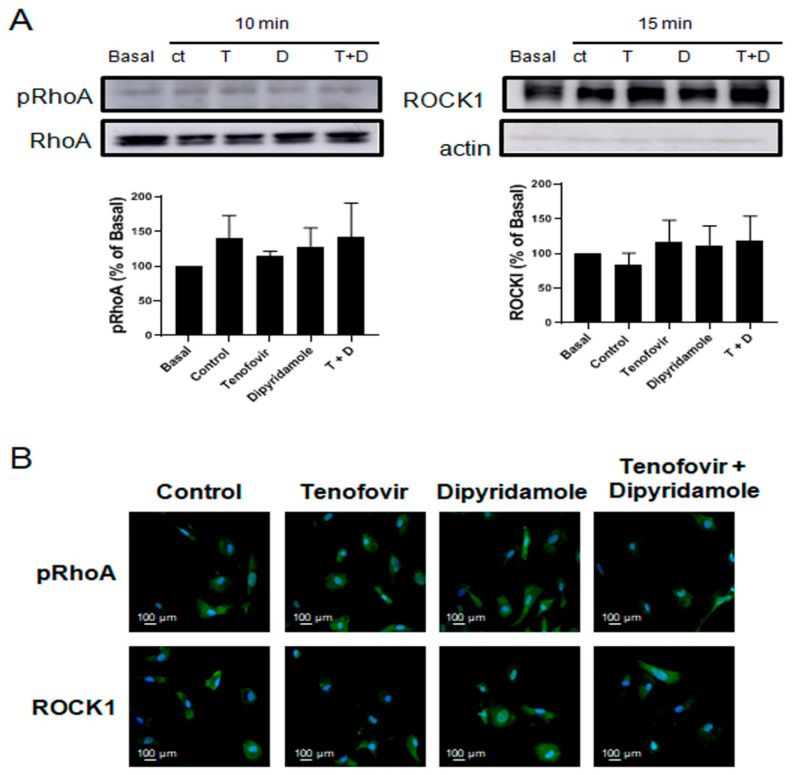
Phosphorylation of RhoA and ROCK1 activation were increased by tenofovir in osteoblast in A2AKO mice. (**A**) pRhoA and ROCK1 expression were studied in osteoblast precursors in the presence of tenofovir at 1 μM alone or with dipyridamole at 1 μM. To normalize for protein loading, the membranes were reprobed with RhoA or actin. (**B**) pRhoA and ROCK1 (green) immunostaining in osteoblast precursors treated with tenofovir at 1 μM alone or with dipyridamole at 1 μM. Nuclei are shown in blue (Dapi). All images were taken at 40× magnification and scale bar indicates 100 µm.

**Figure 6:**
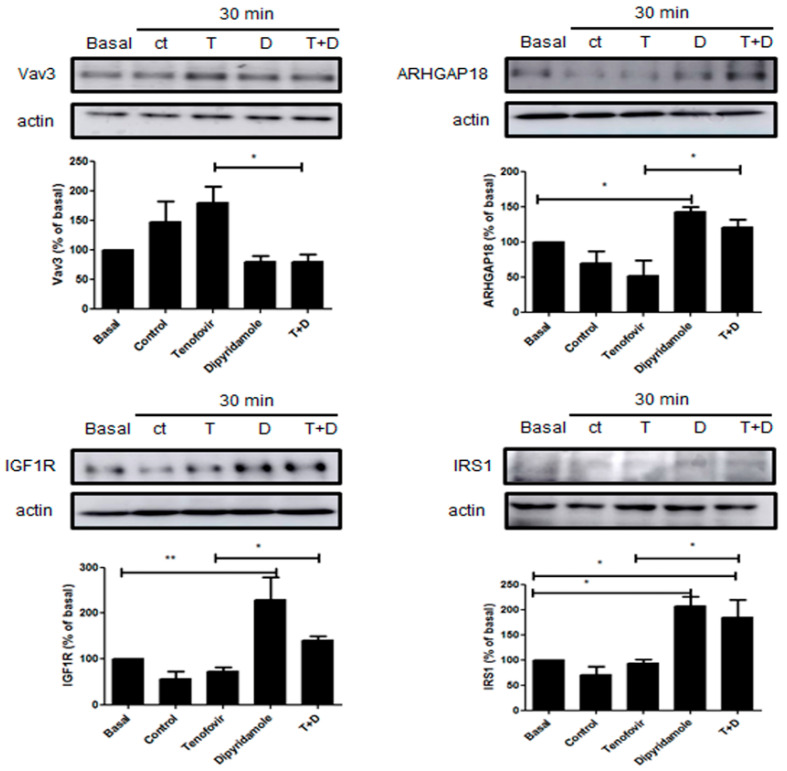
Tenofovir at 1 μM inhibited IGF1R activation via activation of the GEF protein Vav3 in WT mice.Vav3, ARHGAP18, IGF1R and IRS1 expression were studied in osteoblast precursors in the presence of tenofovir at 1 μM alone or with dipyridamole at 1 μM. To normalize for protein loading, the membranes were reprobed with actin. * *p* < 0.05 ** *p* < 0.005 ANOVA.

**Figure 7:**
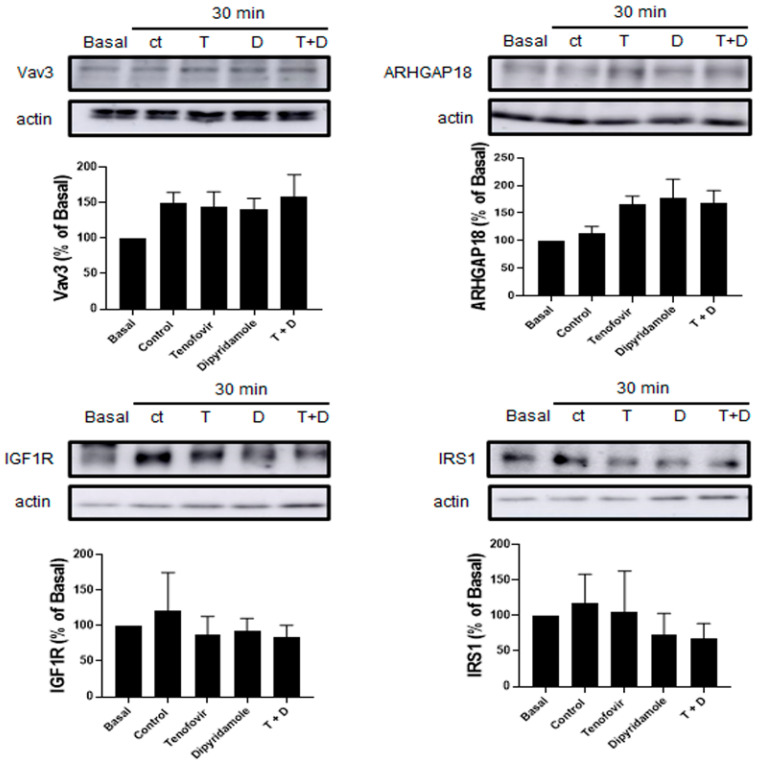
No significant changes in IGF1R activation in A2AKO mice. Vav3, ARHGAP18, IGF1R and IRS1 expression were studied in osteoblast precursors in the presence of tenofovir at 1 μM alone or with dipyridamole at 1 μM. To normalize for protein; the membranes were reprobed with actin.

It has been established that Sema4D/Plexin-B1 activation results in ErbB2 and RhoA-ROCK activation, and the inhibition of IRS-1/IGF1R and consequent inhibition of osteoblast differentiation [4,25]. To establish the direct effect of tenofovir on osteoblast differentiation inhibition, we dissected this pathway in our system in the presence of tenofovir at 1 μM alone or in combination with dipyridamole at 1 μM (Figure 4, Figure 5, Figure 6 and Figure 7). As can be observed in Figure 4A, tenofovir at 1 μM increased RhoA phosphorylation (32 ± 11% increase vs. basal, *p* < 0.05) and ROCK1 activation (83 ± 35% increase vs. basal, *p* < 0.05) at 10 and 15 min, respectively, after osteoblast precursor stimulation, and pre-treatment with dipyridamole at 1 μM reverted this effect. To confirm the Western blot results, immunofluorescence was carried out for pRhoA and ROCK1 (Figure 4B). As can be observed, tenofovir at 1 μM increased pRhoA and ROCK1 staining 15 min after treatment, and this was reverted in the presence of dipyridamole at 1 μM. The effect of dipyridamole in tenofovir reversion was lost in A2AKO osteoblast precursors (Figure 5).

Treatment with tenofovir at 1 μM increased the expression of GEF protein Vav3 30 min after treatment (79 ± 27% increase vs. basal, *p* = ns), with a consequent decrease in GAP protein ARHGAP18 (49 ± 21% increase vs. basal, *p* = ns), which were both significantly reverted by dipyridamole (*p* < 0.05), inhibited IGF1R activation (27 ± 9% decrease vs. basal, *p* = ns) and did not change IRS1 (7 ± 7% decrease vs. basal *p* = ns). Pre-treatment with dipyridamole at 1 μM significantly reverted these effects (*p*< 0.05) (Figure 6). All observed changes in dipyridamole were A2A-receptor-related (Figure 7).

**Figure 8:**
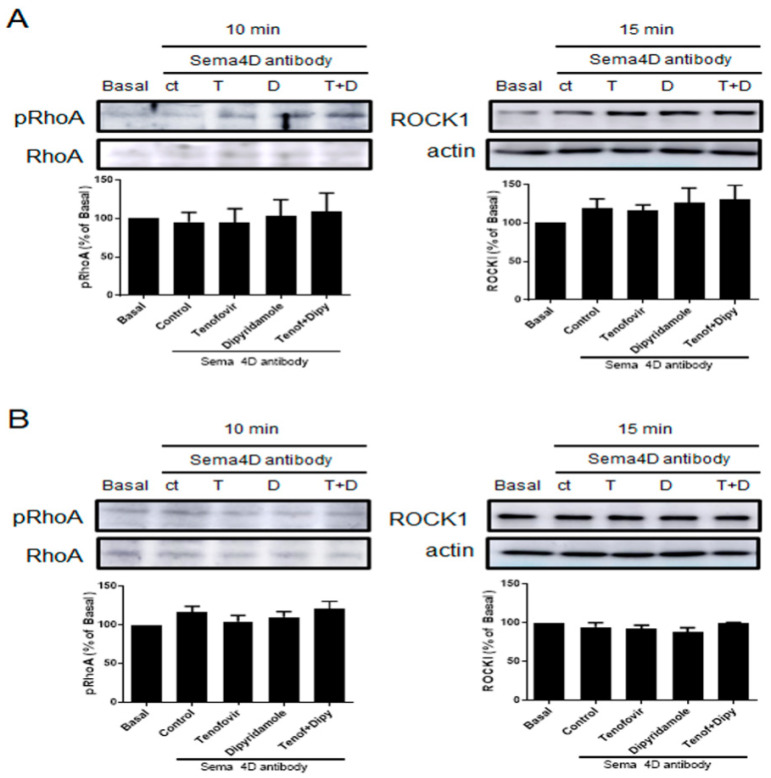
Phosphorylation of RhoA and ROCK1 activation by tenofovir in osteoblast-osteclast co-culture is avoided in the presence of Sema4D antibody. (**A**) pRhoA and ROCK1 expression were studied in WT osteoblast precursors in co-culture the presence of tenofovir at 1 μM and dipyridamole at 1 μM in combination with Sema4D antibody 10 ng/mL. (**B**) pRhoA and ROCK1 expression were studied in A2AKO osteoblast precursors in co-culture the presence of tenofovir at 1 μM and dipyridamole at 1 μM in combination with Sema4D antibody 10 ng/mL. To normalize for protein loading, the membranes were reprobed with RhoA or actin.

To corroborate that Tenofovir exerts the described effects, a murine primary osteoblast-osteoclast precursor co-culture was performed in the presence of Sema4D antibody 10 ng/mL and phosphorylation of RhoA and ROCKI activation were studied by Western blot. In WT mice, in the presence of Sema4D antibody, tenofovir is not able to increase RhoA phosphorylation nor activate ROCKI (Figure 8A). Similar results were observed in A2AKO mice (Figure 8B).

## 3. Discussion

In the last two decades, studies on HIV infection-related bone alterations have increased [26,27,28,29]. Tenofovir is one of the antivirals that showed a greater decrease in BMD [19]. We have described how tenofovir, an HIV drug that inhibits ATP transport and decreases adenosine concentrations, directly activated osteoclast differentiation and function both in vitro and in vivo, and inhibited osteoblast formation, and treatment with compounds that increase extracellular adenosine levels, such as dipyridamole, were able to revert the effect [22].

**Figure 9:**
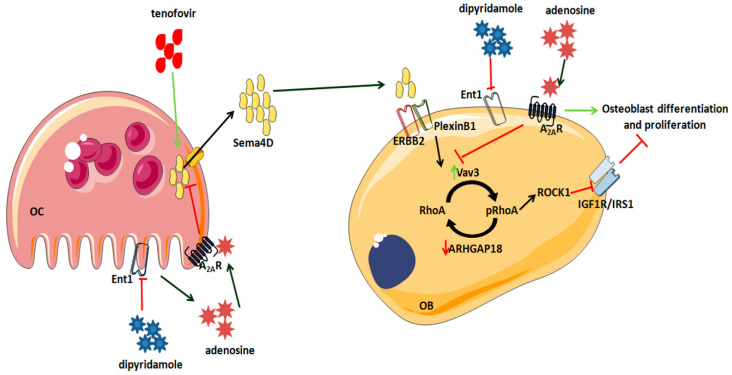
Proposed pathway for tenofovir and Sema4D. Tenofovir promotes Sema4D secretion from osteoclast, and activation of PlexinB1 in osteoblast, activation of RhoA phosphorylation and ROCK1 activation and inhibition of IGF1R/IRS1. Treatment with compounds that increase extracellular adenosine levels, such as dipyridamole, can revert this effect. OC: osteoclast; OB: osteoblast, A2AR: adenosine A2A receptor: Ent1: Equilibrative nucleoside transporter1; Sema 4D: Semaphorin 4D; ERBB2: receptor tyrosine-protein kinase erbB-2. Vav3:Vav Guanine Nucleotide Exchange Factor 3: ARHGAP18: Rho GTPase Activating Protein 18. IGF1: insulin-like growth factor-1; IRS1: Insulin receptor substrate 1.

To maintain the integrity of bones, constant remodeling occurs. Osteoblasts, osteocytes, and osteoclasts synthesize and secrete paracrine signaling molecules, including neurotrophins such as Sema4D [30]. Herein, we suggest that tenofovir directly inhibits osteoblast differentiation, and hence contributes to a decrease in BMD, by activation of the Sema4D/PlexinB1 signaling in osteoclast/osteoblast. We have shown that tenofovir induces the expression and secretion of Sema4D from the osteoclast that leads into a PlexinB1 activation cascade in osteoblast, with changes in RhoA/ROCK1 cytoskeleton and inhibition of the IGF1 signal, and, therefore, inhibits osteoblast differentiation (Figure 9). We have also observed that indirect activation of the adenosine A2AR with dipyridamole reverted these changes. These data correlate with our previous data, where we characterized a crosstalk pathway between adenosine A2AR and Semaphorins [24]. We found that adenosine A2AR activation diminishes the secretion of Sema4D by osteoclasts and enhances the secretion of Sema3A by osteoblasts, leading to an increase in osteoblast differentiation and function. In vivo, we observed that, in combination with the suppressive effects of adenosine A2AR on osteoclast differentiation and function, adenosine A2AR activation diminishes bone osteolysis [24].

As far as we know, this is one of the first studies to ascribe the direct effects of osteoblast intracellular pathway changes to tenofovir treatment. It was recently believed that there were no changes in osteoblast cell viability at clinically relevant tenofovir afelamide (TAF) concentrations and, therefore, osteoblasts do not represent a TAF-sensitive cell type [31]. Moreover, a microarray analysis in primary murine osteoblasts was used to identify the genes altered by TDF [32]. They found that TDF downregulated several genes involved in signal transduction pathways, the cell cycle, amino acid, and energy metabolism, with an overall impact of downregulating osteoblast differentiation and function [32]. Among the downregulated genes, Sema3D was found, but no data were noted on other semaphorin subtypes, or on the intracellular pathways that they modified.

There are no previous data on Sema4D in HIV bone alterations, although a relevant role of Sema4D (CD100) was recently found, in the regulation of the immune system in HIV patients [17]. It was found that CD100 was constitutively expressed on T cells, that CD100/CD72 was required for T-cell proliferation, and CD100 up-regulation at the surface of B cells was associated with B-cell activation and differentiation [33]. The authors found that efficient antiretroviral treatment reverted CD100′s high expression on CD4^+^ and CD8^+^ T cell surfaces, indicating that a replicative virus was needed to induce the up-regulation of CD100. Therefore, they indicate that CD100 and its receptor, CD72, may be another exhaustion marker presented by the immune system. Moreover, observations were made in elite controllers that supported the hypothesis that the preservation of CD100 was important for enhanced viral control and T-cell function in untreated individuals, as CD100Sema4DCD8^+^ T cells counts were lower when compared with noncontrollers [16].

This apparent controversy regarding the role of Sema4D in bone cells, shown in this manuscript and the data on Sema4D and the immune system under HIV treatment needs further study in the future. In the work performed by Correa-Roche et al. [17], the authors did not mention which antiretroviral treatment regimen HIV patients were undergoing, so no data were found on tenofovir. Regardless, it is clear that the mechanisms involved in Semaphorin expression and function in immune and bone cells are different and need to be studied.

In conclusion, our results suggest that tenofovir increases bone loss by activation of Sema4D/PlexinB1 signaling, which inhibits osteoblast differentiation, and treatment with agents that increase local adenosine concentrations, such as dipyridamole, might prevent bone loss following inhibition of the pathway and increases in osteoblast differentiation.

## 4. Materials and Methods

### 4.1. Reagents

Tenofovir was purchased from Sequoia Research Products (Carbosynth Limited, Compton, Berkshire, UK) Recombinant mouse M-CSF and RANKL were obtained from R&D Systems (Minneapolis, MN, USA). α-MEM, FBS and penicillin/streptomycin were from Invitrogen (Life Technologies, New York, NY, USA). Dipyridamole, RIPA buffer, protease inhibitor cocktail, phosphatase inhibitor cocktail, dexamethasone, ascorbic acid and β-glycerophosphate were from Sigma Aldrich (Madrid, Spain). Goat anti-rabbit-FITC was from Invitrogen (Fisher Scientific, Madrid, Spain). Primary antibodies against Sema4D, Plexin B1, RhoA, ARHGAP18, Vav3, IGF1R, IRS-1 were from Abcam (Cambridge, UK). pRhoA primary antibody was Affinity Biosciences (antibodies-online, GmbH). Anti-ROCK1 primary antibody was from Santa Cruz Biotechnology (Dallas, TX, USA).

### 4.2. Mice

All protocols were approved by the Spanish Regulations and the Guidelines for the Care and Use of Laboratory Animals and fulfill international guidelines. The experimental protocol was approved by the Institutional Ethics and Welfare Committee of theIIS-Fundación Jiménez Díaz (Proex 019/19, 14 February 2019).

Female C57Bl/6 and A2AKO mice (12 wk old) were divided into five groups: sham (no surgery), ovariectomized (OVX) saline 0.9% (control), OVX tenofovir 75 mg/Kg/day, OVX dipyridamole 25 mg/Kg/day and OVX combination tenofovir 75 mg/Kg/day and dipyridamole 25 mg/Kg/day (*n* = 5 each). Tenofovir subcutaneous and dipyridamole intraperitoneal were daily injected. Water and food were given ad libitum until sacrifice. Animals were sacrificed after 5 weeks of treatment in a CO_2_ chamber and the long bones were removed, fixed, and prepared for histological staining.

### 4.3. Histological Studies

WT long bones (femur) were fixed in 4% paraformaldehyde for 48 h, followed by decalcification in 10% EDTA for four weeks and paraffin embedding. 5 µm sections were made. Immunohistochemistry analysis for Sema4D and PlexinB1 was carried out as previously described [24]. Briefly, after deparaffination, samples were hydrated and antigen retrieval was performed with Proteinase K solution (20 μg/mL in Tris/EDTA buffer, pH 8.0) for 15 min in a water bath at 37 °C. Blocking of nonspecific binding with PBS 3% BSA and 0.1% Triton X-100 was carried out for 1 h and primary antibodies anti-Sema4D, and PlexinB1 1:200 were incubated overnight at 4 °C in a humidifying chamber. Secondary goat anti-rabbit-FITC (1:200) was incubated for 1 h at room temperature in the dark. After washing, cells were mounted with Fluoroshield with DAPI mounting media (Sigma-Aldrich, Madrid, Spain). Images were observed under a fluorescence microscope (Nikon, Tokio, Japan) equipped with ACT-1 software version 2.63.

### 4.4. Osteoclast Differentiation

BMCs were isolated from 6–8wk-old female C57BL/6 or A2AKO mice, as previously described [22]. The marrow cavity was aseptically flushed out with α-MEM from femora and tibiae, and the marrow was incubated overnight in α-MEM containing 10% FBS and 1% penicillin/streptomycin to obtain a single-cell suspension. A total of 200,000 non-adherent cells were collected and seeded in α-MEM with 30 ng/mLM-CSF for two days. At day 3 (day 0 of differentiation), 30 ng/mLRANKL was added to cultures in the presence/absence of tenofovir 1 µM), alone or in combination with dipyridamole 1 µM (*n* = 5). Medium and reagents were replaced every third day and cells were incubated for 24 h or up to seven days. In all experiments, DMSO was added to control medium.

### 4.5. Osteoblast Differentiation

Osteogenesis assays were performed as previously described [22]. Adherent BMCs from 6–8-wk-old female C57BL/6 or A2AKO mice were seeded at a density of 1×10^5^ cell/cm^2^ density with osteogenic medium (αMEM containing 1 µM dexamethasone, 50 µg/mL ascorbic acid, 10 mM β-glycerophosphate) in the presence/absence of tenofovir 1 µM alone or in combination with dipyridamole 1 µM (*n* = 5) for up to 14 days. In all experiments, DMSO was added to control medium.

### 4.6. Co-Culture of Osteoclast Precursors and Osteoblasts

To determine the involvement of Sema4D signaling in tenofovir mediated effects, we constructed a murine primary osteoblast-osteoclast precursor co-culture system based on previously described protocols [34]. Briefly, co-culture is performed as follows. Adherent BMCs from 6–8-wk-old female C57BL/6 or A2AKO mice (*n* = 5 each) were seeded at a density of 1 × 10^5^ cell/cm^2^ density with osteogenic medium in the bottom chamber of transwell plates (Corning, New York, NY, USA), and atotal of 200,000 non-adherent cells were collected and seeded in α-MEM with 30 ng/mLM-CSF for two days in the top chamber of transwell plates. At day 3 (day 0 of differentiation), 30 ng/mLRANKL was added to cultures in the presence/absence of Sema4D antibody 10 ng/mL alone or in combination with tenofovir or dipyridamole 1 µM each. Cells and supernatant were collected at different time points for western blot analysis (*n* = 5 each).

### 4.7. Western Blot

WT/A2AKO osteoclast or osteoblast precursors were treated with tenofovir and dipyridamole 1 µM each for 10–30 min or 24 h (*n* = 4–5 each). Cells were lysed with RIPA buffer, and the supernatant contents were extracted with cold ethanol–1% SDS and sonicated. Protein concentration was determined by BCA. 4 µg protein (cell lysate) or 40 µg protein (supernatant) was subjected to 7.5 or 10% SDS-PAGE and transferred to a nitrocellulose membrane. Tris-buffered saline/Tween-20 0.05% BSA 3% was used for nonspecific binding. Membranes were incubated overnight (4 °C) with primary rabbit antibodies against Sema4D, PlexinB1, pRhoA, RhoA, ROCK1, Vav3, ARHGAP18 and IGF1R (1:1000 each). Membranes were incubated with goat anti-rabbit HRP 1:5000. Bands were visualized by Amersham Imager 600 (GE Healthcare). Reprobing with actin was performed to check that all lanes were loaded with the same amount of protein. Digital densitometric band analysis was performed using the Quantity One software version 4.6.3 (Bio-Rad, Madrid, Spain), and band intensities were expressed relative to actin or RhoA, as appropriate. EZ blue was used as a loading marker for the supernatant proteins. Variations in intensity were expressed as % of control and expressed as mean ± SEM. All results were calculated as a percentage of non-stimulated controls to minimize the intrinsic variation among different experiments. Statistical analysis was performed by one-way ANOVA and Bonferroni post-test and the levels of significance were indicated in the figure legends.

### 4.8. Real-Time Quantitative RT-PCR

To validate the role of Sema4D in tenofovir signaling, WT/A2AKO primary osteoclast and osteoblast precursors were challenged with tenofovir and dipyridamole 1 µM each for 7 and 14 days, respectively, and mRNA expression of Sema4D/PlexinB1 was analyzed (*n* = 6). Total RNA was extracted using E.Z.N.A. HP total RNA kit (Omega Bio-Tek, VWR International, Norcross, GA, USA) following manufactures protocol. Total RNA was retrotranscribed using MuLV Reverse transcriptase PCR kit (Applied Biosystems, Foster City, CA, USA) at 2.5 U/μL, including the following reagents, in the same reaction: Rnase Inhibitor 1 U/μL (Applied Biosystems), Random Hexamers 2.5 U/μL (Applied Biosystems), MgCl_2_ 5 mM (Applied Biosystems), PCR buffer II 1× (Applied Biosystems) and dNTPs 1mM (Applied Biosystems, Foster City, CA, USA). Relative quantification of gene expression was performed using real-time RT–PCR on a Step One Plus (Applied Biosystems, Foster City, CA, USA) with Power UP SYBR Green MasterMix (Applied Biosystems) according to the manufacturer’s protocol. The following primers were used in real-time PCR amplification: Sema4D forward, 5′-TCTTTGCTGACGTGATCCAG-3′ and reverse, 5′-CAGATCAGCCTGGCCTTTAG-3′; PlexinB1 forward, 5′-TGGGTCATGTGCAGTACGAT-3′ and reverse, 5′-CACTGCTCTCCAGGTTCTCC-3′; and *GAPDH* forward, 5′-CTACACTGAGGACCAGGTTGTCT-3′ and reverse, 5′-GGTCTGGGATGGAAATTGTG-3′. The Pfaffl method [35] was used for relative quantification.

### 4.9. Immunocytochemistry

WT/A2AKO osteoblast precursors were plated in chamber slides and treated with tenofovir 1 µM, alone or in the presence of dipyridamole 1 µM. After 15 min in culture, cells were fixed with 4% paraformaldehyde in PBS, blocked with PBS 3% BSA and 0.1% Triton X-100 for 30 min, and incubated overnight with anti-pRhoA and ROCK1 antibodies. Secondary anti-rabbit FITC was incubated for 1 h in the dark, and slides were mounted with Fluoroshield with DAPI mounting medium (Sigma-Aldrich). Images were observed under a fluorescence microscope (Nikon, Tokyo, Japan), equipped with Nis Elements F3.0 SP7 or ACT-1 software version 2.63.

## 5. Statistical Analysis

The statistical significance of differences among groups was determined by the use of one-way ANOVA and Bonferroni post hoctest. All statistics were calculated using GraphPad software (La Jolla, CA, USA).

## Data Availability

Data is contained within the article.

## References

[1] Edwards C.M., Mundy G.R. (2008). Eph receptors and ephrin signaling pathways: A role in bone homeostasis. Int. J. Med. Sci..

[2] Mediero A., Ramkhelawon B., Perez-Aso M., Moore K.J., Cronstein B.N. (2015). Netrin-1 is a critical autocrine/paracrine factor for osteoclast differentiation. J. Bone Min. Res..

[3] Togari A., Mogi M., Arai M., Yamamoto S., Koshihara Y. (2000). Expression of mRNA for axon guidance molecules, such as semaphorin-III, netrins and neurotrophins, in human osteoblasts and osteoclasts. Brain Res..

[4] Negishi-Koga T., Shinohara M., Komatsu N., Bito H., Kodama T., Friedel R.H., Takayanagi H. (2011). Suppression of bone formation by osteoclastic expression of semaphorin 4D. Nat. Med..

[5] Dacquin R., Domenget C., Kumanogoh A., Kikutani H., Jurdic P., Machuca-Gayet I. (2011). Control of bone resorption by semaphorin 4D is dependent on ovarian function. PLoS ONE.

[6] Boyce B.F. (2013). Advances in the regulation of osteoclasts and osteoclast functions. J. Dent. Res..

[7] Kikutani H., Suzuki K., Kumanogoh A. (2007). Immune semaphorins: Increasing members and their diverse roles. Adv. Immunol..

[8] Maleki K.T., Cornillet M., Björkström N.K. (2016). Soluble SEMA4D/CD100: A novel immunoregulator in infectious and inflammatory diseases. Clin. Immunol..

[9] Ch’ng E.S., Kumanogoh A. (2010). Roles of Sema4D and Plexin-B1 in tumor progression. Mol. Cancer.

[10] Chabbert-de Ponnat I., Marie-Cardine A., Pasterkamp R.J., Schiavon V., Tamagnone L., Thomasset N., Bensussan A., Boumsell L. (2005). Soluble CD100 functions on human monocytes and immature dendritic cells require plexin C1 and plexin B1, respectively. Int. Immunol..

[11] Herold C., Elhabazi A., Bismuth G., Bensussan A., Boumsell L. (1996). CD100 is associated with CD45 at the surface of human T lymphocytes. Role in T cell homotypic adhesion. J. Immunol..

[12] Kumanogoh A., Kikutani H. (2003). Immune semaphorins: A new area of semaphorin research. J. Cell Sci..

[13] Ishida I., Kumanogoh A., Suzuki K., Akahani S., Noda K., Kikutani H. (2003). Involvement of CD100, a lymphocyte semaphorin, in the activation of the human immune system via CD72: Implications for the regulation of immune and inflammatory responses. Int. Immunol..

[14] Wang X., Kumanogoh A., Watanabe C., Shi W., Yoshida K., Kikutani H. (2001). Functional soluble CD100/sema4D released from activated lymphocytes: Possible role in normal and pathologic immune responses. Blood.

[15] Yoshida Y., Ogata A., Kang S., Ebina K., Shi K., Nojima S., Kimura T., Ito D., Morimoto K., Nishide M. (2015). Semaphorin 4D Contributes to Rheumatoid Arthritis by Inducing Inflammatory Cytokine Production: Pathogenic and Therapeutic Implications. Arthritis Rheumatol..

[16] Eriksson E.M., Milush J.M., Ho E.L., Batista M.D., Holditch S.J., Keh C.E., Norris P.J., Keating S.M., Deeks S.G., Hunt P.W. (2012). Expansion of CD8+ T cells lacking Sema4D/CD100 during HIV-1 infection identifies a subset of T cells with decreased functional capacity. Blood.

[17] Correa-Rocha R., Lopez-Abente J., Gutierrez C., Perez-Fernandez V.A., Prieto-Sanchez A., Moreno-Guillen S., Muñoz-Fernandez M.A., Pion M. (2018). CD72/CD100 and PD-1/PD-L1 markers are increased on T and B cells in HIV-1+ viremic individuals, and CD72/CD100 axis is correlated with T-cell exhaustion. PLoS ONE.

[18] Vadasz Z., Elbirt D., Radian S., Bezalel-Rosenberg S., Mahlab-Guri K., Toubi E., Asherb I., Sthoeger Z. (2018). Low levels of the immunoregulator Semaphorin 4D (CD100) in sera of HIV patients. Clin. Immunol..

[19] Brown T.T., Ross A.C., Storer N., Labbato D., McComsey G.A. (2011). Bone turnover, osteoprotegerin/RANKL and inflammation with antiretroviral initiation: Tenofovir versus non-tenofovir regimens. Antivir. Ther..

[20] Feig J.L., Mediero A., Corciulo C., Liu H., Zhang J., Perez-Aso M., Picard L., Wilder T., Cronstein B. (2017). The antiviral drug tenofovir, an inhibitor of Pannexin-1-mediated ATP release, prevents liver and skin fibrosis by downregulating adenosine levels in the liver and skin. PLoS ONE.

[21] Velasquez S., Eugenin E.A. (2014). Role of Pannexin-1 hemichannels and purinergic receptors in the pathogenesis of human diseases. Front. Physiol..

[22] Conesa-Buendía F.M.C., Llamas-Granda P., Larrañaga-Vera A., Wilder T., Largo R., Herrero-Beaumont G., Cronstein B., Mediero A. (2019). Tenofovir Causes Bone Loss via Decreased Bone Formation and Increased Bone Resorption, Which Can Be Counteracted by Dipyridamole in Mice. J. Bone Min. Res..

[23] Ishack S., Mediero A., Wilder T., Ricci J.L., Cronstein B.N. (2015). Bone regeneration in critical bone defects using three-dimensionally printed beta-tricalcium phosphate/hydroxyapatite scaffolds is enhanced by coating scaffolds with either dipyridamole or BMP-2. J. Biomed. Mater. Res. B Appl. Biomater..

[24] Mediero A., Wilder T., Shah L., Cronstein B.N. (2018). Adenosine A2A receptor (A2AR) stimulation modulates expression of semaphorins 4D and 3A, regulators of bone homeostasis. FASEB J..

[25] Kang S., Kumanogoh A. (2013). Semaphorins in bone development, homeostasis, and disease. Semin. Cell Dev. Biol..

[26] Brown T.T., Qaqish R.B. (2006). Antiretroviral therapy and the prevalence of osteopenia and osteoporosis: A meta-analytic review. AIDS.

[27] Erlandson K.M., Allshouse A.A., Jankowski C.M., MaWhinney S., Kohrt W.M., Campbell T.B. (2013). Functional impairment is associated with low bone and muscle mass among persons aging with HIV infection. J. Acquir. Immune Defic. Syndr..

[28] Starup-Linde J., Rosendahl S.B., Storgaard M., Langdahl B. (2020). Management of Osteoporosis in Patients Living With HIV-A Systematic Review and Meta-analysis. J. Acquir. Immune Defic. Syndr..

[29] Parperis K., Abdulqader Y., Myers R., Bhattarai B., Al-Ani M. (2019). Rheumatic diseases in HIV-infected patients in the post-antiretroviral therapy era: A tertiary care center experience. Clin. Rheumatol..

[30] Han Y., You X., Xing W., Zhang Z., Zou W. (2018). Paracrine and endocrine actions of bone—the functions of secretory proteins from osteoblasts, osteocytes, and osteoclasts. Bone Res..

[31] Callebaut C., Liu Y., Babusis D., Ray A., Miller M., Kitrinos K. (2017). Viability of primary osteoblasts after treatment with tenofovir alafenamide: Lack of cytotoxicity at clinically relevant drug concentrations. PLoS ONE.

[32] Grigsby I.F., Pham L., Mansky L.M., Gopalakrishnan R., Carlson A.E., Manskya K.C. (2010). Tenofovir treatment of primary osteoblasts alters gene expression profiles: Implications for bone mineral density loss. Biochem. Biophys. Res. Commun..

[33] Kuklina E.M., Nekrasova I.V., Valieva Y.V. (2017). Involvement of Semaphorin (Sema4D) in T-Dependent Activation of B Cells. Bull. Exp. Biol. Med..

[34] Kruppke B., Ray S., Alt V., Rohnke M., Kern C., Kampschulte M., Heinemann C., Budak M., Adam J., Döhner N. (2020). Gelatin-Modified Calcium/Strontium Hydrogen Phosphates Stimulate Bone Regeneration in Osteoblast/Osteoclast Co-Culture and in Osteoporotic Rat Femur Defects-In Vitro to In Vivo Translation. Molecules.

[35] Pfaffl M.W. (2001). A new mathematical model for relative quantification in real-time RT-PCR. Nucleic Acids Res..

